# Rofecoxib for dysmenorrhoea: meta-analysis using individual patient data

**DOI:** 10.1186/1472-6874-4-5

**Published:** 2004-07-20

**Authors:** Jayne E Edwards, R Andrew Moore, Henry J McQuay

**Affiliations:** 1 Pain Research Unit & Nuffield Department of Anaesthetics University of Oxford The Churchill Headington Oxford OX3 7LJ UK

## Abstract

**Background:**

Individual patient meta-analysis to determine the analgesic efficacy and adverse effects of single-dose rofecoxib in primary dysmenorrhoea.

**Methods:**

Individual patient information was available from three randomised, double blind, placebo and active controlled trials of rofecoxib. Data were combined through meta-analysis. Number-needed-to-treat (NNT) for at least 50% pain relief and the proportion of patients who had taken rescue medication over 12 hours were calculated. Information was collected on adverse effects.

**Results:**

For single-dose rofecoxib 50 mg compared with placebo, the NNTs (with 95% CI) for at least 50% pain relief were 3.2 (2.4 to 4.5) at six, 3.1 (2.4 to 9.0) at eight, and 3.7 (2.8 to 5.6) at 12 hours. For naproxen sodium 550 mg they were 3.1 (2.4 to 4.4) at six, 3.0 (2.3 to 4.2) at eight, and 3.8 (2.7 to 6.1) at 12 hours. The proportion of patients who needed rescue medication within 12 hours was 27% with rofecoxib 50 mg, 29% with naproxen sodium 550 mg, and 50% with placebo. In the single-dose trial, the proportion of patients reporting any adverse effect was 8% (4/49) with rofecoxib 50 mg, 12% (6/49) with ibuprofen 400 mg, and 6% (3/49) with placebo. In the other two multiple dose trials, the proportion of patients reporting any adverse effect was 23% (42/179) with rofecoxib 50 mg, 24% (45/181) with naproxen sodium 550 mg, and 18% (33/178) with placebo.

**Conclusions:**

Single dose rofecoxib 50 mg provided similar pain relief to naproxen sodium 550 mg over 12 hours. The duration of analgesia with rofecoxib 50 mg was similar to that of naproxen sodium 550 mg. Adverse effects were uncommon suggesting safety in short-term use of rofecoxib and naproxen sodium. Future research should include restriction on daily life and absence from work or school as outcomes.

## Background

Dysmenorrhoea is associated with painful cramping of the lower abdominal or back muscles, with or without other symptoms such as nausea, vomiting, and diarrhoea. Onset of dysmenorrhoea is common during adolescence, and up to 50% of women of reproductive age may be affected [1], and 10% incapacitated for up to three days each menstrual cycle. The pain caused by dysmenorrhoea can be debilitating, resulting in women being unable to perform daily activities, and being absent from work or school. In consequence, dysmenorrhoea is associated with emotional, social, and economic burdens. Despite the impact of dysmenorrhoea on daily living, few women seek medical advice [2], or know which treatments work [3].

Raised concentrations of uterine prostaglandins are thought to cause the pain and cramping associated with dysmenorrhoea [4-6]. Nonsteroidal anti-inflammatory drugs (NSAIDs) inhibit prostaglandin synthesis and are commonly used to treat the condition. The newer Cox-2 selective inhibitors (coxibs) also inhibit prostaglandin synthesis, providing an alternative to conventional NSAIDs. Relatively low rates of gastrointestinal adverse effects allow the use of higher doses of coxibs in acute pain and dysmenorrhoea. These high doses may have the additional advantage of longer duration analgesia with extended dosing intervals.

Systematic reviews have shown NSAIDs to be effective in the treatment of primary dysmenorrhoea [7,8] While the latest, Cochrane, review [8] reported on 4,066 women in trials of NSAIDs in dysmenorrhoea, the trials themselves were small, with an average of about 50 women per trial. These 63 randomised double-blind trials investigated 21 different NSAIDS, at different doses, in studies of varying design, varying outcomes, and varying duration.

At least moderate pain relief over a cycle was reported in 14 comparisons between NSAID (any NSAID, any dose) and placebo in 599 women, with an NNT of 2.1 (1.9 to 2.5). The most studied NSAID was naproxen, with 287 women in seven trials, with an NNT of 2.5 (2.0 to 3.3) for this outcome. An earlier review [7] included more trials, some not double-blind, but came to substantially the same conclusion about analgesic efficacy in primary dysmenorrhoea. NSAIDs also improved activities of daily living, though reported in only 216 women [8]. Adverse event information in these trials was not informative given the small number of women and trials, and that adverse events were rare in younger women taking NSAIDs for limited time.

Cox-2 selective inhibitors (coxibs) provide an alternative to conventional NSAIDs, with a potential advantage of once daily dosing [9-11]. This individual patient data meta-analysis of rofecoxib in dysmenorrhoea aimed to determine the efficacy and duration of analgesic activity of single dose rofecoxib, and to evaluate adverse effects.

## Methods

QUOROM guidelines [12] for quality of reporting of meta-analyses were followed though no flow chart was used since trial data all came from a single source.

Merck Research Laboratories, Rahway, New Jersey provided individual patient data from three Phase III trials of rofecoxib in dysmenorrhoea (studies 38, 55 and 56), with the guarantee that all relevant studies completed by July 2002 had been made available. One of the trials has been published in full [9]. Searching PubMed to January 2004 identified one other randomised but open trial of rofecoxib [13] not sponsored by Merck. For inclusion, trials had to be randomised and double blind, compare rofecoxib with placebo, and provide single dose efficacy information. Outcome data were available for pain relief, pain intensity, time to remedication (use of rescue analgesic), and adverse effects. Each trial report was independently read and scored for quality using a three item, 0–5 point scale [14]. For inclusion a trial had to score a minimum of two points, one each for randomisation and double blinding, out of a maximum of 5. A sixteen-point scale was also used to assess trial validity [15].

Our intention was to use two pain outcomes (pain relief over the first dose, and pain relief over the whole cycle) with the outcome being that closest to at least half pain relief. Over the first dose, this might be a measure of total pain relief (TOTPAR), while over a whole cycle it may be a patient global evaluation of good or excellent, rather than mild, fair, no improvement, or worse. Analyses for comparator treatments were based on information available only from the trials of rofecoxib in this report. Outcome data were pooled in an intention to treat (number of patients randomised) analysis. Neither heterogeneity tests nor funnel plots were used [16,17]. Instead clinical homogeneity of trials was examined graphically [18].

Relative benefit (or risk) was calculated using a fixed effects model [19] with no statistically significant difference between treatments assumed when the 95% confidence intervals included 1. Number-needed-to-treat (or harm) was calculated using the method of Cook and Sackett [20] using pooled observations. NNT is the reciprocal of the absolute risk reduction or increase; for instance, if 75 out of 100 patients benefit with treatment and only 25 out of 100 benefit with placebo, the absolute risk increase is 0.75–0.25 = 0.5, and the NNT is 1/0.5 = 2. The z test [21] was used to determine statistical differences between NNTs for different doses, treatments or outcomes. Mean adverse event rates were calculated, weighting by treatment group size. Use of rescue medication was analysed as the proportion of patients remedicating at different time points within 12 hours.

## Results

The mean age of women in the trials was 31 years, and at baseline pain was moderate in 66% of women and severe in 34%. All trials were randomised, double blind, and compared oral doses of rofecoxib with an active control and placebo in women with moderate to severe pain due to dysmenorrhoea. One trial was a single dose crossover (study 38), and two were multiple dose crossovers (studies 55 and 56), where the crossover was between single doses in different menstrual cycles. Single dose efficacy data were available for the first 12 hours of treatment in all trials, but not summary estimates over a cycle. Study designs and quality and validity scores are shown in Table 1. All trials scored the maximum five points for quality and at least 13/16 points for trial validity; some of the criteria for validity were not appropriate because of the individual patient presentation of results.

**Table 1:** Trial details

**Trial ID**	**Study drug and dose, number of women**	**Design**	**Observations after 8 hrs**	**Quality score**	**Validity score**
38	49 women Rofecoxib 25 mg Rofecoxib 50 mg Ibuprofen 400 mg Placebo	Single oral dose, parallel 3 menstrual cycles	12, 24	5/5	≥13/16
55	60 women Rofecoxib 50 mg then 25 mg as required Naproxen sodium 550 mg every 12 hrs Placebo	Oral. Multiple dose study with single dose efficacy data, multiple dose adverse events Cross-over, 1 of 6 drug sequences 3 menstrual cycles	12 hour obervations after a single dose in a three-day study	5/5	≥13/16
56	122 women Rofecoxib 50 mg as required Rofecoxib 50 mg then 25 mg as required Naproxen sodium 550 mg every 12 hrs Placebo	Oral. Multiple dose study with single dose efficacy data, multiple dose adverse events Cross-over, 1 of 4 drug sequences 4 menstrual cycles	12 hour obervations after a single dose in a three-day study	5/5	≥13/16

The single dose trial (study 38) was conducted over three cycles. Each of 49 women received three of four treatments (rofecoxib 25 mg, rofecoxib 50 mg, ibuprofen 400 mg, or placebo). For the two multiple dose studies, one (study 55) compared a single dose of rofecoxib 50 mg followed by 25 mg daily as required, naproxen sodium 550 mg every 12 hours, or placebo in 60 women over three menstrual cycles. The other (study 56) compared rofecoxib 50 mg as required, rofecoxib 50 mg followed by 25 mg daily as required, naproxen sodium 550 mg every 12 hours, or placebo in 122 women over four menstrual cycles. Both trials reported multiple dose adverse effects. In the multiple dose trials, all women received each treatment regimen for one cycle.

Pain intensity and pain relief were measured using the standard 4-point categorical pain intensity scale (0 none, 1 mild, 2 moderate, 3 severe) and a 5-point point pain relief scale (0 none, 1 a little, 2 some, 3 a lot, 4 complete). Pain measurements were collected using patient diaries. Patients were assessed at baseline, then at least hourly for eight hours, and again at 12 hours for single dose efficacy data. The exact time at which a patient requested remedication (or rescue analgesic), if required, was recorded. Adverse effects were recorded as the number of patients with any adverse effect(s), or particular adverse effects.

### Efficacy

Full efficacy results over six, eight and 12 hours are shown in Table 2. All active treatments were significantly more effective than placebo at all time points.

**Table 2:** Number needed to treat for at least 50% pain relief

		**Improved with**		**% improved**			
				
**Number of trials**	**Drug and dose (mg)**	**Active**	**Placebo**	**Active**	**Placebo**	**Relative risk (95% CI)**	**NNT (95% CI)**
**Six hour ourcomes**							
1	Rofecoxib 25	66/115	45/118	57	38	1.5 (1.1 to 2.0)	5.0 (3.7 to 7.8)
3	Rofecoxib 50	140/226	70/225	62	31	2.0 (1.6 to 2.5)	3.2 (2.4 to 4.5)
1	Ibuprofen 400	31/49	10/47	63	21	3.0 (1.7 to 5.4)	2.4 (1.7 to 4.2)
2	Naproxen sodium 550	120/181	60/178	66	34	2.0 (1.6 to 2.5)	3.1 (2.4 to 4.4)
**Eight hour ourcomes**							
1	Rofecoxib 25	70/115	44/118	61	37	1.6 (1.2 to 2.2)	4.2 (2.8 to 9.0)
3	Rofecoxib 50	147/226	73/225	65	32	2.0 (1.6 to 2.5)	3.1 (2.4 to 9.0)
1	Ibuprofen 400	30/47	11/47	61	21	2.6 (1.5 to 4.6)	2.6 (1.8 to 5.1)
2	Naproxen sodium 550	121/181	62/178	68	35	2.0 (1.6 to 2.4)	3.0 (2.3 to 4.3)
**Twelve hour ourcomes**							
1	Rofecoxib 25	64/115	45/118	56	38	1.5 (1.1 to 1.9)	5.7 (3.3 to 20)
3	Rofecoxib 50	135/226	74/225	60	33	1.8 (1.5 to 2.3)	3.7 (2.8 to 5.6)
1	Ibuprofen 400	27/49	12/47	55	26	2.2 (1.3 to 3.7)	3.4 (2.1 to 9.2)
2	Naproxen sodium 550	111/181	62/178	61	35	1.8 (1.4 to 2.2)	3.8 (2.7 to 6.1)

Rofecoxib 25 mg was tested in a single trial, rofecoxib 50 mg in three, ibuprofen 400 mg in one and naproxen sodium 550 mg in two. For all the active analgesics the proportion of patients with at least 50% pain relief was about 60% at all time points, and with placebo it was about 30% at all time points (Table 2). Numbers needed to treat tended to be much the same for rofecoxib 50 mg, ibuprofen 400 mg and naproxen sodium 550 mg, though somewhat higher (worse) for rofecoxib 25 mg (Table 2).

There was no significant difference between NNTs for single doses of study treatments at six, eight or 12 hours. For instance, no significant difference at the 12 hour comparison was seen between the NNTs of rofecoxib 25 mg and rofecoxib 50 mg (z score 1.19, p = 0.23), ibuprofen 400 mg (z score 0.28, p = 0.78), or naproxen sodium 550 mg(z score 1.09, p = 0.28), or between NNTs of rofecoxib 50 mg and ibuprofen 400 mg (z score 0.26, p = 0.79), or naproxen sodium 550 mg (z score 0.096, p = 0.60).

### Remedication

**Figure 1:**
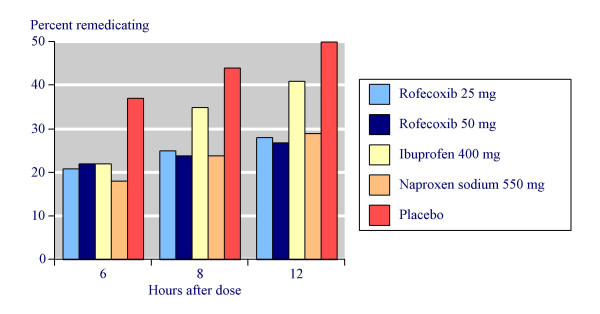
Remedication time for all drugs

The proportion of patients who remedicated at different time points over 12 hours is shown in Figure 1. At 12 hours remedication occurred with 29% on rofecoxib 25 mg, 28% on rofecoxib 50 mg, 29% on naproxen sodium 550 mg, 41% on ibuprofen 400 mg, and 50% with placebo.

### Adverse effects

Few adverse effects of a particular type were reported, and none were serious in any trial. The most commonly reported adverse effects were nausea and somnolence, but these occurred infrequently. A single dose in one trial (study 38) gave the proportion of patients reporting any adverse effect(s) as 10% (5/49 patients) with rofecoxib 25 mg, 8% (4/49) with rofecoxib 50 mg, 12% (6/49) with ibuprofen 400 mg, and 6% (3/49) with placebo. With multiple doses over a cycle, the proportion of patients reporting any adverse effect(s) was 23% (42/179 patients) with rofecoxib 50 mg, 24% (45/181) with naproxen sodium 550 mg, and 18% (33/178) with placebo.

## Discussion

Pain with dysmenorrhoea usually lasts for about three days, though with considerable individual variation. Trials of analgesics can have various forms. The simplest might be to give the same analgesic for the whole of the painful cycle, and ask a global question concerning efficacy at the end. Women might then be crossed over to a different treatment at the next cycle. A variation would be to use the same basic structure, but make more detailed evaluations of pain or pain relief over a limited time during the first day, though a global question could always be added. A more complicated design would use a cross-over within a single cycle.

The three trials described here used a cross-over between cycles, with detailed pain measurements over 12–24 hours in the first painful day. Twelve-hour and 24-hour outcomes have also been reported in two other recent studies of coxibs in dysmenorrhoea [10,11]. With previous NSAID studies [8] the outcome most often used in placebo-controlled trials was at least moderate pain relief or an equivalent outcome over a whole cycle. A recent open-label study had a crossover design with drugs given each successive day [13].

All three trials were of the highest reporting quality, and had high validity scores, indicating that known sources of bias are unlikely to occur [22,8]. We know that to be sure of a result (as an NNT) we need information from about 400 patients when the NNT is about 2, but much more when the NNT is higher (worse) [23]. Here we have information from about 450 women with rofecoxib 50 mg and 360 with naproxen sodium 550 mg, but only about 200 women contributed for rofecoxib 25 mg and fewer than 100 for ibuprofen 400 mg (Table 2). For rofecoxib 25 mg and ibuprofen 400 mg, therefore, uncertainty over the size of the effect continues.

Individual patient information from three trials of high quality showed that for the outcome of at least half pain relief over 12 hours, rofecoxib, naproxen sodium and ibuprofen were similarly effective in the treatment of pain associated with dysmenorrhoea. This confirms what was known from previous meta-analysis [8], in which most information was for naproxen at various doses with an NNT of 2.5 (2.0 to 3.3) for the outcome of at least moderate pain relief over 3–5 days compared with placebo. In this analysis the NNT for a single dose of 550 mg naproxen sodium was between 3.0 and 3.8 over six to 12 hours. The Cochrane review had information on 287 women in seven placebo-controlled trials, while two of the three trials here had information on 359 women taking naproxen sodium.

Rofecoxib 50 mg was statistically indistinguishable from naproxen sodium 550 mg (Table 2) at all times, though rofecoxib 25 mg tended to have numerically higher (worse) NNTs at all times. Remedication over 12 hours was statistically indistinguishable between rofecoxib doses and naproxen. Here the number of women studied was even larger, with 451 women involved in the trials comparing rofecoxib 50 mg and placebo. The difference between rofecoxib 50 mg and naproxen sodium 550 mg would be in dosing schedules, with once versus twice a day dosing.

Pain relief and duration of analgesia are not the only issues of importance in dysmenorrhoea. The impact of dysmenorrhoea on activities of daily living, disability or function, and absence from work or school are additional factors to be considered. These outcomes were not addressed by the trials for rofecoxib, which were conducted for regulatory purposes. This limits the utility of the information, but trials of other coxibs (also conducted for regulatory purposes) have also concentrated on pain relief and duration of analgesia [10,11].

Future trials should examine a range of short-term analgesia and longer outcomes like interference with daily living or absence from work or school. The analysis by Zhang and colleagues [7] did examine these additional outcomes, and found daily life to be less restricted with naproxen or ibuprofen than with placebo, and fewer absences from work or school to occur with naproxen than with placebo. These outcomes are infrequently reported [8], but are likely to be associated with pain, so decreased pain should improve these other outcomes as well. Verification of this assumption with data from high quality clinical trials would be welcome, though.

Future individual patient analysis of trials in dysmenorrhoea would have the potential to examine issues around the efficacy of analgesics in women with heavy menstrual loss, or who use combined oral contraceptive pills. In this analysis information was not available for these analyses, and in any event any sub-groups would probably have been too small for any definitive answer.

In the trials for rofecoxib, information on adverse effects was collected using diaries. Few adverse effects were reported to have occurred and none were serious. The most common adverse effects were nausea and somnolence. These and headache have been frequently reported with other coxibs [10,11] and NSAIDs [7,24]. The problem when interpreting information on adverse effects, though, is that any symptom can be recorded as an adverse event however tenuous its association to the study drug. We cannot be certain whether these symptoms were due to the condition or to the drug.

## Conclusions

Based on information from three trials, a single dose of rofecoxib 50 mg is as effective as a single dose of naproxen sodium 500 mg in controlling the pain associated with dysmenorrhoea, and causes relatively few adverse effects.

## Competing interests

RAM has been a consultant for Merck, Sharpe and Dohme Ltd, UK. RAM, JE and HJM have received lecture fees from pharmaceutical companies. The authors have received research support from charities and government sources at various times, but no such support was received for this work. Neither author has any direct stock holding in any pharmaceutical company. The terms of the financial support from MSD included freedom for authors to reach their own conclusions, and an absolute right to publish the results of their research, irrespective of any conclusions reached. MSD did have the right to view the final manuscript before publication, and did so.

## Authors' contributions

JE conducted the analyses, which were checked by RAM. All authors contributed equally to the design, writing and reviewing of the paper.

**Table 3:** Proportion of women who used rescue analgesic

	**Percent who remedicated by (hrs)**		
	
**Drug & dose (mg)**	6	8	12
Rofecoxib 25	21	25	28
Rofecoxib 50	22	24	27
Ibuprofen 400	22	35	41
Naproxen sodium 550	18	24	29
Placebo	37	44	50

## Pre-publication history

The pre-publication history for this paper can be accessed here:



## References

[B1] Dawood MY (1988). Nonsteroidal anti-inflammatory drugs and changing attitudes toward dysmenorrhoea. American Journal of Medicine.

[B2] Hewison A, van den Akker OB (1996). Dysmenorrhoea, menstrual attitude and GP consultation. British Journal of Nursing.

[B3] Hillen TI, Grbavac SL, Johnston PJ, Straton JA, Keogh JM (1999). Primary dysmenorrhoea in young Western Australian women: prevalence, impact and knowledge of treatment. Journal of Adolescent Health.

[B4] Dawood MY (1993). Nonsteroidal antiinflammatory drugs and reproduction. American Journal of Obstetrics and Gynecology.

[B5] Bieglmayer C, Hofer G, Kainz C, Reinthaller A, Kopp B, Janisch H (1995). Concentrations of various arachidonic acid metabolites in menstrual fluid are associated with menstrual pain and are influenced by hormonal contraceptives. Gynecological Endocrinolology.

[B6] Nigam S, Benedetto C, Zonca M, Leo-Rossberg I, Lubbert H, Hammerstein J (1991). Increased concentrations of eicosanoids and platelet-activating factor in menstrual blood from women with primary dysmenorrhea. Eicosanoids.

[B7] Zhang WY, Li Wan Po A (1998). Efficacy of minor analgesics in primary dysmenorrhoea: a systematic review. British Journal of Obstetrics and Gynaecology.

[B8] Marjoribanks J, Proctor M, Farquhar C (2003). Nonsteroidal anti-inflammatory drugs for primary dysmenorrhoea. Cochrane Database of Systematic Reviews.

[B9] Morrison BW, Daniels SE, Kotey P, Cantu N, Seidenberg B (1999). Rofecoxib, a specific cyclooxygenase-2 inhibitor, in primary dysmenorrhea: a randomized controlled trial. Obstetrics & Gynecology.

[B10] Daniels SE, Talwalker S, Torri S, Snabes MC, Recker DP, Verburg KM (2002). Valdecoxib, a cyclooxygenase-2-specific inhibitor, is effective in treating primary dysmenorrhea. Obstetrics & Gynecology.

[B11] Malmstrom K, Kotey P, Cichanowitz N, Daniels S, Desjardins PJ (2003). Analgesic efficacy of etoricoxib in primary dysmenorrhea: results of a randomized, controlled trial. Gynecology Obstetrics Investigations.

[B12] Moher D, Cook DJ, Eastwood S, Olkin I, Rennie D, Stroup DF (1999). Improving the quality of meta-analyses of randomised controlled trials: the QUOROM statement. Lancet.

[B13] Sahin I, Saracoglu F, Kurban Y, Turkkani B (2003). Dysmenorrhea treatment with a single daily dose of rofecoxib. International Journal of Gynaecology and Obstetrics.

[B14] Jadad AR, More RA, Carroll D, Jenkinson C, Reynolds DJM, Gavaghan DJ, McQuay HJ (1996). Assessing the quality of reports of randomised clinical trials: is blinding necessary?. Controlled Clinical Trials.

[B15] Smith LA, Oldman AD, McQuay HJ, Moore RA (2000). Teasing apart quality and validity in systematic reviews: an example from acupuncture trials in chronic neck and back pain. Pain.

[B16] Gavaghan DJ, Moore RA, McQuay HJ (2000). An evaluation of homogeneity tests in meta-analyses in pain using simulations of individual patient data. Pain.

[B17] Tang J-L, Liu JLY (2000). Misleading funnel plot for detection of bias in meta-analysis. Journal of Clinical Epidemiology.

[B18] L'Abbé KA, Detsky AS, O'Rourke K (1987). Meta-analysis in clinical research. Annals of International Medicine.

[B19] Morris JA, Gardner MJ, Gardner MJ, Altman DG (1995). Calculating confidence intervals for relative risk, odds ratios and standardised ratios and rates. Statistics with confidence – confidence intervals and statistical guidelines.

[B20] Cook RJ, Sackett DL (1995). The number needed to treat: a clinically useful measure of treatment effect. British Medical Journal.

[B21] Tramèr MR, Reynolds DJM, Moore RA, McQuay HJ (1997). Impact of covert duplicate publication on meta-analysis: a case study. British Medical Journal.

[B22] Moore RA, Gavaghan D, Tramer MR, Collins SL, McQuay HJ (1998). Size is everything-large amounts of information are needed to overcome random effects in estimating direction and magnitude of treatment effects. Pain.

[B23] Khan KS, Daya S, Jadad AR (1996). The importance of quality of primary studies in producing unbiased systematic reviews. Arch Intern Med.

[B24] Milsom I, Minic M, Dawood MY, Akin MD, Spann J, Niland NF, Squire RA (2002). Comparison of the efficacy and safety of nonprescription doses of naproxen and naproxen sodium with ibuprofen, acetaminophen, and placebo in the treatment of primary dysmenorrhea: a pooled analysis of five studies. Clinical Therapeutics.

